# Leading Causes of Death among Asian American Subgroups (2003–2011)

**DOI:** 10.1371/journal.pone.0124341

**Published:** 2015-04-27

**Authors:** Katherine G. Hastings, Powell O. Jose, Kristopher I. Kapphahn, Ariel T. H. Frank, Benjamin A. Goldstein, Caroline A. Thompson, Karen Eggleston, Mark R. Cullen, Latha P. Palaniappan

**Affiliations:** 1 Stanford University School of Medicine, Division of General Medical Disciplines, Stanford, California, United States of America; 2 Sutter Health Medical Foundation, Department of Cardiology, Davis, California, United States of America; 3 Columbia University School of Nursing, New York, New York, United States of America; 4 Palo Alto Medical Foundation Research Institute, Palo Alto, California, United States of America; 5 Stanford University, Shorenstein Asia-Pacific Research Center, Stanford, California, United States of America; California Department of Public Health, UNITED STATES

## Abstract

**Background:**

Our current understanding of Asian American mortality patterns has been distorted by the historical aggregation of diverse Asian subgroups on death certificates, masking important differences in the leading causes of death across subgroups. In this analysis, we aim to fill an important knowledge gap in Asian American health by reporting leading causes of mortality by disaggregated Asian American subgroups.

**Methods and Findings:**

We examined national mortality records for the six largest Asian subgroups (Asian Indian, Chinese, Filipino, Japanese, Korean, Vietnamese) and non-Hispanic Whites (NHWs) from 2003-2011, and ranked the leading causes of death. We calculated all-cause and cause-specific age-adjusted rates, temporal trends with annual percent changes, and rate ratios by race/ethnicity and sex. Rankings revealed that as an aggregated group, cancer was the leading cause of death for Asian Americans. When disaggregated, there was notable heterogeneity. Among women, cancer was the leading cause of death for every group except Asian Indians. In men, cancer was the leading cause of death among Chinese, Korean, and Vietnamese men, while heart disease was the leading cause of death among Asian Indians, Filipino and Japanese men. The proportion of death due to heart disease for Asian Indian males was nearly double that of cancer (31% vs. 18%). Temporal trends showed increased mortality of cancer and diabetes in Asian Indians and Vietnamese; increased stroke mortality in Asian Indians; increased suicide mortality in Koreans; and increased mortality from Alzheimer’s disease for all racial/ethnic groups from 2003-2011. All-cause rate ratios revealed that overall mortality is lower in Asian Americans compared to NHWs.

**Conclusions:**

Our findings show heterogeneity in the leading causes of death among Asian American subgroups. Additional research should focus on culturally competent and cost-effective approaches to prevent and treat specific diseases among these growing diverse populations.

## Introduction

As one of the fastest growing racial/ethnic groups in the U.S., the Asian Americans population is expected to double in size from18 million in 2010 to 34 million in 2050.[1][3] Certain Asian subgroups have seen dramatic increases in population sizes since 2000, ranging from a 20% increase for Koreans to 50% increase for Asian Indians.[2] However, our current understanding of Asian American health is largely based on aggregated data [3–6], and very little is known about the individual Asian subgroups which vary in disease occurrence, immigration patterns, socioeconomic backgrounds, and dietary and cultural practices. The six largest Asian American subgroups in the U.S. are Asian Indians, Chinese, Filipinos, Japanese, Koreans, and Vietnamese; these subgroups account for 84% of all Asian Americans in the U.S.[1] Major federal surveys which are used to set the national agenda for disparities research (e.g., the National Health and Nutrition Examination Survey) currently do not adequately sample Asian Americans, nor do they report data by Asian American subgroup (although there are plans to do so in the future).[7] Given the limited data reported on individual subgroups, President Obama in 2009 signed an Executive Order calling for strategies to improve the health of Asian Americans and to seek data on the health disparities among Asian American subgroups.[8]

The aggregation of Asian Americans masks important differences in mortality among subgroups. To date, the majority of death record data available in the U.S. is from California, where several studies have suggested important differences in cause-specific mortality, with Asian Indians having higher mortality due to coronary heart disease[9, 10] and Chinese and Japanese having higher stroke[10] and cancer[11]mortality. Prior to implementation of the 2003 Standard U.S. death certificate, reporting of decedent race on death certificates used a fill-in-the-blank approach. Federal instructions on how to complete this fill-in-the blank field, including a list of specific race categories, became more granular over time. Starting in 1992, Chinese, Japanese, and Filipino were suggested race categories in all states. The additional categories of Asian Indian, Korean, and Vietnamese were suggested for collection in 7 states most populated with these ethnicities. The 2003 Standard U.S death certificate provides a check box for each of the six largest Asian subgroups. Since the 2003 standard became available, 36 U.S. states and the District of Columbia have adopted it as of 2011(year of adoption varies by state, with 5 of the previously mentioned 7 most populous states adopting the new standard by 2004).[12–16] While the degree to which states disaggregated Asian subgroups varied across our study period, we believe the adoption of the 2003 standard corresponds to an increase in accuracy of subgroup reporting.[17] The U.S. Census began disaggregating Asian subgroups in 1980, which provides self reported racial/ethnic data to calculate population sizes.

Immigration history differs among Asian subgroups, and has not only shaped geographic concentration, but also demographic characteristics and socioeconomic disparities between subgroups, and may also play an important role in observed mortality differences.[3] Brief immigration histories for individual Asian subgroups have been outlined in a previous publication.[3] Based on the American Community Survey (ACS) 2011 data shown in Table 1, nativity, educational attainment, and household income level also varies by Asian subgroup.[2] Prior research has shown that socioeconomic and cultural factors are strongly associated with mortality in other racial groups.[18, 19]

**Table 1:** 2011 Demographics of the six largest single race Asian subgroups and Non-Hispanic Whites in the United States.^1^

Subgroup	Total Population (margin of error)	Foreign-Born Population (%)	Education (Bachelor’s Degree or higher^2^) (%)	Median Household Income ($) (margin of error)
NHW	197,084, 523 (+/- 22,989)	7,599,684 (3.9%)	31.9%	55,305 (+/-104)
Asian Indian	2,908,204 (+/- 52,033)	2,071,853 (71.2%)	32.6%	92,418 (+/-1,820)
Chinese	3,520,150 (+/-44,000)	2,451,814 (69.7%)	24.9%	63,999 (+/-1,442)
Filipino	2,538,325 (+/-47,496)	1,688,391 (66.5%)	39.4%	78,700 (+/-1,772)
Japanese	756,898 (+/-18,355)	293,457 (38.8%)	31.4%	66,146 (+/-2,114)
Korean	1,449,876 (+/-30,304)	1,066,171 (73.5%)	34.7%	51,625 (+/-1,264)
Vietnamese	1,669,447 (+/-40,719)	1,119,126 (67.0%)	18.4%	54,670 (+/-1,950)

^1^Data from 2011 American Community Survey 1-Year Estimates;

^2^Based on the population ≥25 years of age

There is currently a knowledge gap in the health of these rapidly expanding populations with little evidence to recommend research agendas, create public health policy, and offer clinical guidelines. The White House,[8] the American Heart Association,[3] and the National, Heart, Lung and Blood Institute[13] have all acknowledged the paucity of data and the need for research about diverse Asian American subgroups. Therefore, it is important to examine subgroup mortality trends to inform the national agenda for disparities research. The adoption of the most recent revision of the death certificate, which collects coded mortality data by Asian subgroup for over half the U.S. states beginning in 2003, enables us to do so with more accuracy and coverage than the previous death certificate version. In this analysis, we aim to fill an important knowledge gap in Asian American health by reporting all-cause and cause-specific mortality by disaggregated subgroups.

## Materials and Methods

### Study Data

We examined the U.S. mortality records from the National Center for Health Statistics’ (NCHS) Multiple Cause of Death mortality files from 2003–2011 for Asians and non-Hispanic Whites (NHWs) in 36 states and the District of Columbia. Decedents represent the following Asian ethnicities: Asian Indian, Chinese, Filipino, Japanese, Korean or Vietnamese. Any decedents reported as more than one Asian race, Asian Hispanic, “Other Asian”, were excluded from this analysis. The 36 states were selected based on their adoption of the 2003 revision of the U.S. Standard Certificate of Death during the study period: Arizona, Arkansas, California, Connecticut, Delaware, Florida, Georgia, Idaho, Illinois, Indiana, Iowa, Kansas, Kentucky, Maine, Michigan, Minnesota, Missouri, Montana, Nebraska, Nevada, New Hampshire, New Jersey, New Mexico, New York (including New York City), North Dakota, Ohio, Oklahoma, Oregon, Rhode Island, South Carolina, South Dakota, Texas, Utah, Vermont, Washington, and Wyoming.

Year of death, age, location, race/ethnicity of the decedent and the underlying cause of death (disease or injury that initiated the events resulting in death) were identified from death certificate data obtained from the National Center for Health Statistics (NCHS). “Underlying cause of death” was coded by NCHS using International Classification of Diseases, 10th revision (ICD-10). Race/ethnicity was recorded on death certificates by the funeral director using national guidelines and coded to the 7 categories of interest (NHW, Asian Indian, Chinese, Filipino, Japanese, Korean or Vietnamese) by the NCHS. We selected only the six Asian subgroups represented explicitly in both the 2003 U.S. Standard Certificate of Death and U.S. Census forms. We also excluded individuals classified as more than one Asian race because they account for a very small portion of all Asian Americans within our study population.

In order to estimate denominator population counts for the study period, we examined trends in the total U.S. population by racial/ethnic groups using American Community Survey (ACS) data and found that all groups grew linearly from 2000 to 2010.[2] We then investigated the accuracy of interpolation with ACS data stratified by age group and race and found that interpolated values corresponded well to observed values, particularly for the oldest age groups. Therefore, for each race/ethnicity and sex group, we used the population data from the 2000 and 2010 census to estimate population counts for non-Census years. Specifically, we fit a line over time using the 2000 and 2010 census counts as observed endpoints and used the values along this line as population estimates for years 2003–2011(study period). The ranked leading causes of death, death counts, and percentages of death are presented for NHWs, Aggregate Asians (combined group of six Asian subgroups), and each Asian subgroup population by sex.

### Statistical Analysis

**Fig 1:**
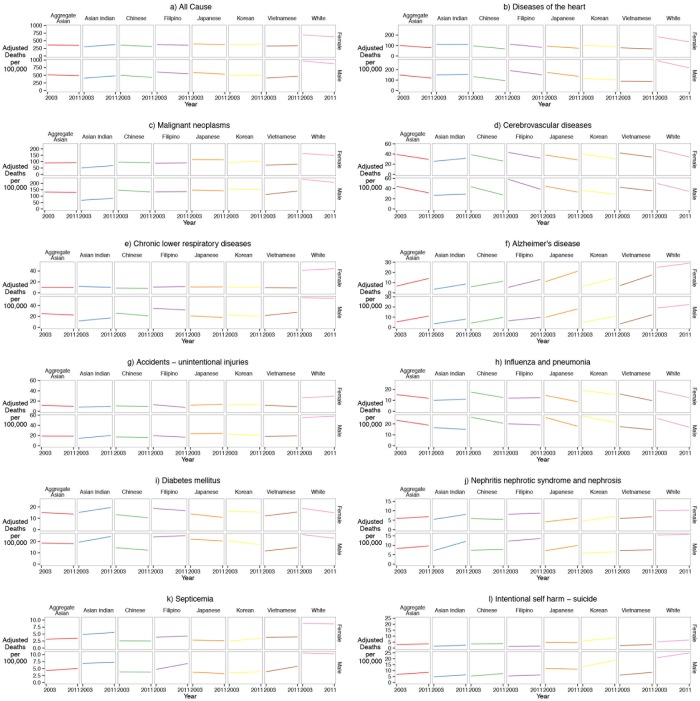
Temporal trends (2003–2011) of all-cause and cause-specific mortality rates for Asian American subgroups and NHWs by sex in 36 U.S states and the District of Columbia.

We first determined the leading causes of death for NHW and each Asian subgroup by sex. Causes of death were ranked according to death counts as recommended by the 1951 Public Health Conference on Records and Statistics and the National Vital Statistics Reports published by NCHS.[6] We used the same cause of death categories as the 1951 recommendations, which includes 50 major causes of death, aggregating categories for similar causes (i.e. “Diseases of the Heart” or “Malignant Neoplasms”). We calculated age-adjusted mortality rates and adjusted rate ratios by year and overall using direct standardization for age adjustment to the 2000 US census population. For overall rates, annual death counts for each age, race, sex, and cause were averaged across the 2003–2011 time period and divided by the estimated 2007 population as previously described (the approximate midpoint of the time period) producing age, race, sex, cause-specific raw mortality rates. Rate ratios used total NHW population for the reference category. We calculated standardized rate differences (SRD) by subtracting cause-specific adjusted rates for NHW from cause-specific adjusted rates for each subgroup. The SRDs for all cause mortality were decomposed into percent attributable to each individual cause by dividing the individual cause SRD by the all cause SRD. To examine trends in mortality, we graphed age-adjusted mortality rates by racial/ethnic group, cause of death, and sex (Fig 1). Joinpoint regression models and average annual percentage change (AAPC) statistics with 95% confidence intervals were used to characterize the magnitude and direction of trends. Data management and analyses were conducted using R version 3.1.0, Ruby 1.9.3p194 and MySQL 5.6.17.

## Results

The study population included 13,208,036 NHW and Asian decedents collected from 36 states during 2003 to 2011. All-cause age-adjusted mortality rates and rate ratios were overall higher for NHWs compared to “Aggregate Asians” and each of the disaggregated subgroups (Table 2). The ranked leading causes of death were determined by death counts for the leading causes of death for each Asian subgroup and NHW by sex (Table 3). Malignant neoplasms (cancer) were the leading cause of death (28.6% of all deaths) in Asian American females collectively (labeled “Aggregate Asian”) and for all Asian female subgroups, except for Asian Indians. Diseases of the Heart (heart disease) rank as the leading cause of death for Asian Indian females as well as NHW females. Cause-specific proportions of deaths by subgroup revealed variation in the relative frequency of cancer and heart disease for Korean (30% vs. 22% respectively), Vietnamese (28% vs. 19% respectively), Japanese (29% vs. 23% respectively), and Asian Indian females (23% vs. 28% respectively). Cerebrovascular disease (stroke) ranked third among all females. The rankings for causes beyond the top three varied by subgroup for Asian females. Chronic lower respiratory disease is a much less common cause of death in Asian American females, compared to NHW females. Conversely, diabetes ranked as a more important cause of death in Asian females, particularly Asian Indians (5% of all deaths) and Filipinas (5% of all deaths).

**Table 2:** Total number of deaths, age-adjusted mortality rates, and rate ratios (RR) from all causes by racial/ethnic group and sex in the United States, 2003–2011 (36 States and District of Columbia).

Female	NHW	Aggregate Asian	Asian Indian	Chinese	Filipino	Japanese	Korean	Vietnamese
Total # Deaths	6,608,844	124,600	13,118	37,166	30,806	18,960	14,196	10,354
Population Size [1]	73,030,653	4,908,967	976,037	1,375,599	1,084,108	300,131	579,867	593,225
Adjusted mortality rate [2]	658.9	353.3	340.8	330.2	363.3	388.8	381.8	330.7
Adjusted Rate Ratio	1.0	0.54	0.52	0.5	0.55	0.59	0.58	0.5
**Male**	**NHW**	**Aggregate Asian**	**Asian Indian**	**Chinese**	**Filipino**	**Japanese**	**Korean**	**Vietnamese**
Total # Deaths	6,342,612	131,980	19,270	41,418	30,694	14,435	12,675	13,488
Population Size[1]	70,544,365	4,429,486	1,064,888	1,232,121	863,474	219,570	476,646	572,786
Adjusted mortality rate[2]	921.0	499.5	446.7	462.3	574.9	561.8	495.7	441.2
Adjusted Rate Ratio	1.0	0.54	0.49	0.5	0.62	0.61	0.54	0.48

**Table 3:** Rankings (Rk), death count, and percentage of death due to cause (%) by racial/ethnic group and sex, from 2003–2011 (36 States and District of Columbia).

Female	NHW			Aggregate Asian			Asian Indian			Chinese			Filipino			Japanese			Korean			Vietnamese		
Cause of Death	Rk	Count	%	Rk	Count	%	Rk	Count	%	Rk	Count	%	Rk	Count	%	Rk	Count	%	Rk	Count	%	Rk	Count	%
**All Causes**		6,608,844	100		124,600	100		13,118	100		37,166	100		30,806	100		18,960	100		14,196	100		10,354	100
**Diseases of heart**	1	1,705,306	25.8	2	29,294	23.5	1	3,651	27.8	2	8,795	23.7	2	7,519	24.4	2	4,289	22.6	2	3,060	21.6	2	1,980	19.1
**Malignant neoplasms**	2	1,451,773	22	1	35,582	28.6	2	2,952	22.5	1	11,156	30	1	8,854	28.7	1	5,421	28.6	1	4,307	30.3	1	2,892	27.9
**Cerebrovascular diseases**	3	442,875	6.7	3	11,526	9.3	3	947	7.2	3	3,497	9.4	3	3,068	10	3	1,670	8.8	3	1,233	8.7	3	1,111	10.7
**Chronic lower respiratory diseases**	4	421,534	6.4	7	3,217	2.6	7	322	2.5	8	914	2.5	7	834	2.7	7	563	3	7	338	2.4	8	246	2.4
**Alzheimer’s Disease**	5	312,547	4.7	8	3,183	2.6	9	155	1.2	7	933	2.5	9	633	2.1	4	872	4.6	8	306	2.2	7	284	2.7
**Accidents-Unintentional injuries**	6	223,733	3.4	6	4,080	3.3	5	481	3.7	6	1,158	3.1	5	911	3	8	523	2.8	4	600	4.2	4	407	3.9
**Influenza and pneumonia**	7	166,547	2.5	5	4,277	3.4	6	343	2.6	4	1,594	4.3	6	906	2.9	6	587	3.1	6	537	3.8	6	310	3
**Diabetes Mellitus**	8	164,077	2.5	4	4,934	4	4	631	4.8	5	1,274	3.4	4	1,469	4.8	5	611	3.2	5	552	3.9	5	397	3.8
**Nephritis, nephrotic syndrome and nephrosis**	9	106,140	1.6	9	2,197	1.8	8	232	1.8	10	619	1.7	8	700	2.3	10	260	1.4	10	199	1.4	10	187	1.8
**Septicemia**	10	86,787	1.3	10	1,167	0.9	11	188	1.4	12	282	0.8	11	337	1.1	15	136	0.7	12	109	0.8	12	115	1.1
**Male**	**NHW**			**Aggregate Asian**			**Asian Indian**			**Chinese**			**Filipino**			**Japanese**			**Korean**			**Vietnamese**		
**Cause of Death**	**Rk**	**Count**	**%**	**Rk**	**Count**	**%**	**Rk**	**Count**	**%**	**Rk**	**Count**	**%**	**Rk**	**Count**	**%**	**Rk**	**Count**	**%**	**Rk**	**Count**	**%**	**Rk**	**Count**	**%**
**All Causes**		6,342,612	100		131,980	100		19,270	100		41,418	100		30,694	100		14,435	100		12,675	100		13,488	100
**Diseases of heart**	1	1,713,546	27	2	33,148	25.1	1	6,025	31.3	2	9,629	23.2	1	8,647	28.2	1	4,011	27.8	2	2,431	19.2	2	2,405	17.8
**Malignant neoplasms**	2	1,573,285	24.8	1	37,043	28.1	2	3,477	18	1	13,312	32.1	2	7,847	25.6	2	3,687	25.5	1	4,340	34.2	1	4,380	32.5
**Chronic lower respiratory diseases**	3	372,517	5.9	5	5,243	4	8	427	2.2	4	1,896	4.6	4	1,451	4.7	7	510	3.5	8	403	3.2	5	556	4.1
**Accidents-Unintentional injuries**	4	368,914	5.8	4	6,194	4.7	3	1,254	6.5	6	1,645	4	6	1,191	3.9	4	569	3.9	4	753	5.9	4	782	5.8
**Cerebrovascular diseases**	5	280,776	4.4	3	9,269	7	4	993	5.2	3	2,986	7.2	3	2,457	8	3	1,004	7	3	775	6.1	3	1,054	7.8
**Diabetes Mellitus**	6	172,964	2.7	6	4,804	3.6	5	914	4.7	7	1,173	2.8	5	1,367	4.5	6	551	3.8	6	438	3.5	7	361	2.7
**Intentional self-harm (suicide)**	7	152,732	2.4	8	3,064	2.3	6	550	2.9	8	735	1.8	13	487	1.6	9	265	1.8	5	635	5	6	392	2.9
**Influenza and pneumonia**	8	136,415	2.2	7	4,532	3.4	7	470	2.4	5	1,844	4.5	7	881	2.9	5	566	3.9	7	436	3.4	8	335	2.5
**Alzheimer’s Disease**	9	133,028	2.1	10	1,608	1.2	10	123	0.6	11	529	1.3	10	306	1	8	382	2.6	10	130	1	10	138	1
**Nephritis, nephrotic syndrome, and nephrosis**	10	104,836	1.7	9	2,168	1.6	9	328	1.7	9	636	1.5	8	647	2.1	10	224	1.6	11	144	1.1	8	189	1.4

Source: National Center for Health Statistics: Diseases of the heart (International Classification of Diseases- 10^th^ revision [ICD-10] codes I00-I09, I11, I13, I20-I51); Malignant Neoplasms (C00-C97); Chronic lower respiratory diseases (J40-J47); Accidents-unintentional injury (V01-X59, Y85-Y86); Cerebrovascular disease (I60-I69); Diabetes Mellitus (E10-E14); Intentional self-harm (suicide) (U03, X60-X84, Y87.0); Influenza and pneumonia (J09-J18); Alzheimer’s Disease (G30); Nephritis, nephrotic syndrome, and nephrosis (N00-N07, N17-N19, N25-N27); Septicemia (A40-A41).

For males, patterns between the proportions of deaths due to heart disease and cancer differed from those found in females. Cancer was the leading cause of death for aggregated Asian males (27.4%); however when disaggregated, cancer is the leading cause of death only for Chinese, Korean, and Vietnamese males (Table 3). Heart disease was the leading cause of death for Filipino, Japanese, and Asian Indian males. Notably, the proportions of death due to heart disease for Asian Indian males were nearly double that of cancer (31% vs. 18% respectively). There were also marked and reverse differences between heart disease and cancer mortality, respectively, among Korean (19% vs. 34%) and Vietnamese (18% vs. 33%) males. Overall, stroke ranked as the third leading cause of death among male Asian subgroups. Similar to Asian females, diabetes mellitus ranked higher among leading causes of death for Asian males than for NHW males. Korean males had more than twice the frequency of death due to suicide (5%) compared to NHWs (2%) and suicide was ranked as the fifth leading cause of death, higher than every other Asian subgroup and NHWs. Mortality rates for suicide among Korean males were twice that of any other Asian subgroup (Table 4).

**Table 4:** Age-adjusted mortality rates (AR) per 100,000 by cause of death, racial/ethnic group, and sex: 36 U.S. States and District of Columbia, 2003–2011 average.

Female	NHW	Aggregate Asian	Asian Indian	Chinese	Filipino	Japanese	Korean	Vietnamese
Cause of Death	AR^1^	AR	AR	AR	AR	AR	AR	AR
Diseases of the heart	158.5	88.5	111.8	80.5	95.9	83.3	93.8	72.3
Malignant neoplasms	155.8	91.2	60.4	93.7	89.1	115.9	98.9	76.5
Cerebrovascular diseases	41.2	33.7	28.9	31.7	36.9	32.9	34.7	37.5
Chronic lower respiratory diseases	42.6	9.9	10.8	8.4	11.0	10.9	10.3	9.3
Alzheimer’s Disease	27.0	10.3	6.0	8.6	9.2	16.2	10.3	12.2
Accidents-Unintentional injuries	27.8	10.5	8.8	9.9	9.8	12.6	13.1	10.2
Influenza and pneumonia	15.4	13.3	10.6	14.7	12.1	11.1	16.9	12.2
Diabetes Mellitus	16.7	14.3	17.5	11.7	17.6	12.3	15.8	13.9
Nephritis, nephrotic syndrome, and nephrosis	10.2	6.4	6.8	5.6	8.56	5.2	5.8	6.4
Septicemia	8.7	3.3	5.2	2.5	4.1	2.7	2.9	3.9
**Male**	**NHW**	**Aggregate Asian**	**Asian Indian**	**Chinese**	**Filipino**	**Japanese**	**Korean**	**Vietnamese**
**Cause of Death**	**AR**	**AR**	**AR**	**AR**	**AR**	**AR**	**AR**	**AR**
Diseases of heart	248.7	131.9	152.8	111.3	168.9	152.9	107.7	87.6
Malignant neoplasms	219.1	130.5	75.9	139.3	133.8	145.1	153.9	127.1
Chronic lower respiratory diseases	53.8	23.7	14.6	23.3	33.2	19.6	21.2	24.9
Accidents-Unintentional injuries	56.5	18.9	17.4	16.7	18.1	24.0	21.5	19.0
Cerebrovascular diseases	41.6	37.1	28.1	34.2	47.4	38.1	32.2	38.5
Diabetes Mellitus	24.4	18.3	22.1	13.3	24.5	21.2	18.7	13.2
Intentional self-harm (suicide)	22.9	7.7	5.7	6.5	6.0	11.5	15.9	7.5
Influenza and pneumonia	20.6	20.8	15.6	22.9	19.5	21.4	23.8	15.8
Alzheimer’s Disease	20.7	8.2	5.8	6.9	8.0	14	7.8	7.5
Nephritis, nephrotic syndrome, and nephrosis	15.6	8.9	9.8	7.6	12.9	8.6	6.2	7.4

^1^Age-adjusted mortality rates standardized to 2000 US standard population

Cause-specific age-adjusted mortality rates for the leading causes of death were generally higher for NHWs compared to all Asian subgroups (Table 4). However, there was substantial heterogeneity in cause-specific mortality between sexes for each of the Asian subgroups. Heart disease rates were highest for Asian Indian females (111.8 per 100,000 population) compared to all other Asian subgroups. As for males, Filipinos (168.9) experienced the highest heart disease mortality rates compared to other Asian subgroups. Japanese females (115.9) had the highest cancer rates and Asian Indian females (93.7) had the lowest rates compared to all other Asian counterparts. Cancer mortality rates were highest for Korean (153.9) and lowest for Asian Indian males (75.9). Both Asian Indian females (28.9) and males (28.1) had the lowest stroke mortality, whereas Vietnamese females (37.5) and Filipino males (47.4) presented the highest compared to other Asian subgroups (Table 4). In Table 5, SRD’s are presented to capture mortality rate differences by Asian subgroup in comparison to NHW mortality rates.

**Table 5:** Decomposition of standardized rate differences (SRD) by all-cause and cause-specific mortality among Asian American subgroups over the 9-year study period (2003–2011), and percentage of change in all-cause adjusted rate difference attributable to each cause of death.

Females														
Cause of Death	Aggregate Asian		Asian Indian		Chinese		Filipino		Japanese		Korean		Vietnamese	
	SRD^1^	%	SRD	%	SRD	%	SRD	%	SRD	%	SRD	%	SRD	%
All Cause	-438.8	100	-460.7	100	-472.8	100	-422.6	100	-393.4	100	-382.9	100	-479.2	100
Diseases of the heart	-106.1	24.2	-70.8	15.4	-119.3	25.2	-92.1	21.8	-113.7	28.9	-92.5	24.2	-136.6	28.5
Malignant neoplasms	-89	20.3	-134.4	29.2	-83.9	17.7	-94.8	22.4	-56.4	14.3	-76.3	19.9	-110.9	23.1
Cerebrovascular diseases	-11.8	2.7	-18.6	4	-14.8	3.1	-6.9	1.6	-13	3.3	-9.4	2.5	-6.3	1.3
Chronic lower respiratory diseases	-47.2	10.8	-45.8	9.9	-49.4	10.4	-44.6	10.5	-47.2	12	-45.7	11.9	-48	10
Alzheimer’s disease	-28.1	6.4	-36.3	7.9	-31.4	6.6	-29.7	7.0	-17.4	4.4	-28.3	7.4	-24.9	5.2
Accidents—unintentional injuries	-19.6	4.5	-21.8	4.7	-20.3	4.3	-20.9	4.9	-16.8	4.3	-16.6	4.3	-20.1	4.2
Influenza and pneumonia	-1.6	0.4	-7.2	1.6	0.5	-0.1	-3.3	0.8	-5.2	1.3	5.6	-1.5	-4.1	0.9
Diabetes mellitus	-1.8	0.4	2.6	-0.6	-5.4	1.1	3.3	-0.8	-6.1	1.5	0.8	-0.2	-2.2	0.5
Nephritis, nephrotic syndrome, and nephrosis	-5.6	1.3	-5.2	1.1	-6.7	1.4	-2.2	0.5	-8	2	-6.3	1.6	-5.9	1.2
Septicemia	-7.9	1.8	-4.9	1.1	-9	1.9	-6.8	1.6	-9.1	2.3	-8.3	2.2	-6.9	1.4
**Males**														
**Cause of Death**	**Aggregate Asian**		**Asian Indian**		**Chinese**		**Filipino**		**Japanese**		**Korean**		**Vietnamese**	
	**SRD**	**%**	**SRD**	**%**	**SRD**	**%**	**SRD**	**%**	**SRD**	**%**	**SRD**	**%**	**SRD**	**%**
All Cause	-463.4	100	-507.9	100	-506.9	100	-385.6	100	-397.3	100	-459.7	100	-517.5	100
Diseases of the heart	-129	27.8	-99.5	19.6	-152.8	30.1	-91.3	23.7	-106.5	26.8	-154.3	33.6	-175	33.8
Malignant neoplasms	-101.7	21.9	-161.8	31.9	-92.8	18.3	-97.6	25.3	-85.9	21.6	-72.7	15.8	-102.2	19.7
Chronic lower respiratory diseases	-34.2	7.4	-43.1	8.5	-34.8	6.9	-24.5	6.4	-38.2	9.6	-36.5	7.9	-32.7	6.3
Accidents—unintentional injuries	-38.3	8.3	-39.5	7.8	-40.7	8.0	-39.3	10.2	-33.1	8.3	-35.1	7.6	-38.2	7.4
Cerebrovascular diseases	-4.6	1.0	-13.6	2.7	-7.8	1.5	6.4	-1.7	-4	1	-9.1	2	-2.2	0.4
Diabetes mellitus	-7.1	1.5	-2.4	0.5	-13	2.6	0.1	0	-4.3	1.1	-6.9	1.5	-12.7	2.5
Intentional self harm—suicide	-15.8	3.4	-17.9	3.5	-17	3.4	-17.9	4.6	-11.5	2.9	-6.4	1.4	-16.3	3.1
Influenza and pneumonia	-0.4	0.1	-5	1.0	1.7	-0.3	-1.7	0.4	0.5	-0.1	2.9	-0.6	-5.1	1.0
Alzheimer’s disease	-12.9	2.8	-14.9	2.9	-14.2	2.8	-13.2	3.4	-7	1.8	-13.2	2.9	-13.2	2.6
Nephritis, nephrotic syndrome, and nephrosis	-7.0	1.5	-5.9	1.2	-8.6	1.7	-2.8	0.7	-7.5	1.9	-9.8	2.1	-8.5	1.6

^1^SRDs were calculated by subtracting cause-specific adjusted rates for NHW from cause-specific adjusted rates for each Asian subgroup. The SRDs for all cause mortality were decomposed into percent attributable to each cause by dividing the individual cause SRD by the all-cause SRD.

Temporal trends with average annual percent changes (AAPC; %) showed an overall decrease in cause-specific mortality rates among NHWs, and increasing rates for some Asian subgroups, particularly Asian Indians (Fig 1a–1l; Table 6). Note that confidence intervals are wide, which may weaken statistically significant trends. Trends for all-cause (1a) showed that rates increase for Asian Indian females (AAPC: 2.86; 95% CI: 1.4–4.3) and males (AAPC: 2.26; 95% CI: 1.0–3.5) and Vietnamese males (AAPC: 1.77; 95% CI: 0.5–3.0). Heart disease has decreased for all racial/ethnic groups, but remains steady for Asian Indian males (AAPC: 0.45; 95% CI: -1.6–2.5). Cancer mortality rates are rising for Asian Indian females (AAPC: 3.77; 95% CI: 0.4–7.3) and males (AAPC: 2.52; 95% CI -0.4–5.6), and Vietnamese males (AAPC: 2.67; 95% CI: 0.4–5.0), but decreasing for Chinese males (AAPC: -1.33; 95% CI -3.4–0.8). Stroke mortality trends decreased at high rates for all racial/ethnic groups, except for Asian Indian females (AAPC: 2.67; 95% CI: -2.1–7.7) and males (AAPC: 1.12; 95% CI: -3.6–6.1). Diabetes rates are also decreasing for majority of racial/ethnic groups, with the exception of Asian Indian females (AAPC: 3.23; 95% CI: -2.9–9.8) and males (AAPC: 2.96; 95% CI: -2.5–8.8), and Vietnamese females (AAPC: 3.12; 95% CI: -3.7–10.5) and males (AAPC: 2.96; 95% CI: -4.0–10.5). Lastly, another important trend to highlight are the increasing rates of suicidal mortality (1l) (AAPC: 4.34; 95% CI: -2.1–11.2) for Korean males.

**Table 6:** Average annual percent change (AAPC) with 95% confidence intervals for temporal trends by racial/ethnic group, cause of death, and sex for years 2003–2011 using 36 state data.

Females																
Cause of Death	NHW		Aggregate Asian		Asian Indian		Chinese		Filipino		Japanese		Korean		Vietnamese	
	AAPC	95% CI	AAPC	95% CI	AAPC	95% CI	AAPC	95% CI	AAPC	95% CI	AAPC	95% CI	AAPC	95% CI	AAPC	95% CI
All Cause	**-1.11** *	-2.09, -0.14	-0.50	-1.83, 0.85	**2.86** *	1.44, 4.29	**-1.71** *	-3.07, -0.34	-0.93	-2.23, 0.40	-0.55	-1.82, 0.74	0.73	-0.57, 2.04	0.35	-1.04, 1.75
Diseases of the heart	**-3.82** *	-5.74, -1.86	**-3.02** *	-5.59, -0.39	-0.02	-2.39, 2.40	**-4.22** *	-6.88, -1.49	**-3.73** *	-6.18, -1.22	**-2.98** *	-5.63, -0.26	-2.06	-4.58, 0.53	-1.85	-4.73, 1.10
Malignant neoplasms	-1.16	-3.14, 0.86	0.33	-2.30, 3.03	**3.77** *	0.42, 7.25	-0.59	-3.15, 2.04	0.39	-2.26, 3.13	-0.18	-2.51, 2.20	1.28	-1.27, 3.90	1.25	-1.64, 4.24
Cerebrovascular diseases	**-4.29** *	-8.01, -0.43	-3.47	-7.59, 0.80	2.67	-2.07, 7.67	**-4.81** *	-8.99, -0.46	-3.68	-7.61, 0.40	-3.55	-7.72, 0.79	-3.33	-7.39, 0.89	-2.59	-6.52, 1.50
Chronic lower respiratory diseases	0.90	-2.94, 4.89	-0.19	-7.94, 8.21	-1.73	-9.02, 6.11	-0.68	-8.99, 8.37	0.89	-6.53, 8.92	0.22	-7.21, 8.24	0.11	-7.49, 8.33	-0.38	-8.34, 8.25
Alzheimer’s disease	1.88	-2.96, 6.98	**9.88** *	1.31, 19.37	**11.87** *	0.50, 24.97	8.51	-0.64, 18.71	**11.71** *	2.42, 22.13	**8.62** *	1.81, 15.99	**10.95** *	2.26, 20.61	**12.06** *	3.90, 21.06
Accidents—unintentional injuries	1.71	-3.06, 6.72	-2.20	-9.58, 5.75	1.60	-6.77, 10.76	-1.57	-9.22, 6.68	-6.58	-13.90, 1.24	1.46	-5.52, 8.98	1.58	-5.30, 8.98	-3.19	-10.59, 4.76
Influenza and pneumonia	-4.94	-10.94, 1.40	-3.10	-9.60, 3.83	1.28	-6.33, 9.54	-4.08	-10.22, 2.43	0.34	-6.70, 7.92	-6.10	-12.99, 1.23	-2.82	-8.60, 3.31	-5.85	-12.44, 1.15
Diabetes mellitus	-2.90	-8.75, 3.30	-1.22	-7.61, 5.60	3.23	-2.87, 9.75	-2.94	-9.88, 4.49	-1.42	-7.19, 4.69	-3.09	-9.89, 4.17	-0.60	-6.74, 5.94	3.12	-3.70, 10.47
Nephritis, nephrotic syndrome, and nephrosis	0.28	-7.37, 8.57	1.81	-7.91, 12.62	4.98	-4.82, 15.94	-0.79	-10.86, 10.39	0.89	-7.54, 10.10	4.94	-6.28, 17.70	5.75	-4.90, 17.80	1.75	-7.98, 12.57
Septicemia	-0.36	-8.55, 8.57	1.24	-11.91, 16.44	1.85	-8.86, 13.89	-0.24	-15.00, 17.06	1.18	-10.77, 14.78	-1.00	-15.24, 15.55	4.16	-10.19, 21.09	0.38	-11.72, 14.15
**Males**																
**Cause of Death**	**NHW**		**Aggregate Asian**		**Asian Indian**		**Chinese**		**Filipino**		**Japanese**		**Korean**		**Vietnamese**	
	**AAPC**	**95% CI**	**AAPC**	**95% CI**	**AAPC**	**95% CI**	**AAPC**	**95% CI**	**AAPC**	**95% CI**	**AAPC**	**95% CI**	**AAPC**	**95% CI**	**AAPC**	**95% CI**
All Cause	**-1.30** *	-2.12, -0.48	-0.81	-1.92, 0.32	**2.26** *	1.03, 3.50	**-1.82** *	-2.97, -0.66	**-1.28** *	-2.31, -0.23	**-1.24** *	-2.29, -0.18	-0.06	-1.19, 1.08	**1.77** *	0.54, 3.01
Diseases of the heart	**-3.25** *	-4.79, -1.68	**-2.75** *	-4.87, -0.60	0.45	-1.59, 2.53	**-4.13** *	-6.40, -1.81	**-2.95** *	-4.82, -1.05	**-2.96** *	-4.93, -0.96	-1.36	-3.73, 1.07	-0.52	-3.17, 2.20
Malignant neoplasms	-1.37	-3.04, 0.33	-0.19	-2.38, 2.04	2.52	-0.43, 5.57	-1.33	-3.42, 0.81	0.16	-2.01, 2.37	-0.56	-2.63, 1.55	-0.31	-2.33, 1.74	**2.67** *	0.38, 5.02
Chronic lower respiratory diseases	-0.20	-3.59, 3.30	-1.29	-6.29, 3.97	4.97	-1.84, 12.32	-2.59	-7.57, 2.64	-1.11	-5.36, 3.32	-1.74	-7.22, 4.05	-0.99	-6.28, 4.59	3.10	-2.05, 8.54
Accidents—unintentional injuries	0.65	-2.68, 4.10	-0.09	-5.75, 5.92	4.40	-1.81, 11.04	-0.76	-6.72, 5.57	-2.09	-7.74, 3.90	0.26	-4.81, 5.60	-1.47	-6.70, 4.05	0.79	-4.93, 6.86
Cerebrovascular diseases	**-4.51** *	-8.20, -0.70	**-4.15** *	-8.05, -0.11	1.12	-3.61, 6.09	**-5.66** *	-9.66, -1.51	**-4.94** *	-8.38, -1.39	-3.70	-7.57, 0.31	-2.81	-7.04, 1.60	-2.19	-6.08, 1.86
Diabetes mellitus	-1.65	-6.57, 3.52	-0.37	-6.10, 5.70	2.96	-2.48, 8.73	-2.02	-8.60, 5.01	0.56	-4.45, 5.84	-0.97	-6.27, 4.62	-2.10	-7.66, 3.78	2.96	-4.01, 10.49
Intentional self harm—suicide	2.19	-3.08, 7.77	2.84	-6.15, 12.77	4.01	-6.51, 15.85	4.02	-5.83, 15.02	1.91	-8.13, 13.10	-0.60	-7.75, 7.09	4.34	-2.10, 11.25	3.91	-5.34, 14.18
Influenza and pneumonia	-4.72	-9.92, 0.73	-2.59	-7.85, 2.94	-1.24	-7.36, 5.27	-2.88	-7.87, 2.36	-0.62	-6.16, 5.24	-4.52	-9.61, 0.83	-2.95	-7.85, 2.18	-2.13	-8.15, 4.25
Alzheimer’s disease	2.03	-3.51, 7.89	9.49	-0.01, 20.15	11.21	-0.32, 24.51	**11.45** *	0.86, 23.51	5.36	-3.71, 15.41	**7.82** *	0.62, 15.64	**10.66** *	0.77, 21.81	**17.69** *	6.57, 30.54
Nephritis, nephrotic syndrome, and nephrosis	0.20	-6.04, 6.85	1.91	-6.37, 10.96	6.76	-1.71, 16.10	0.84	-8.03, 10.59	1.37	-5.54, 8.80	4.19	-4.51, 13.78	1.37	-8.48, 12.32	0.55	-8.41, 10.40

Bolded* AAPCs indicate statistical significance (p≤0.05).

Supplemental data was included for 50-state data (S1, S2 and S3 Tables). We conducted several sensitivity analyses to explore effects of inclusion of various states on our results. We recalculated rates and rate ratios using all 50 states plus D.C. and again using only CA, FL, HI, IL, NJ, TX, VA and WA (the 7 states reporting all 6 subgroups since 1992). For the 50-state and 7-state subsets, there were no substantive or systematic differences in estimates by subgroup and cause of death when compared to the 36-state results. We additionally examined death counts by year, state, and subgroup to evaluate potential bias introduced by the gradual adoption of the new death certificate standard. We found that all 36 states reported Chinese, Japanese, and Filipino deaths for the entire study period, and that the 7 states mentioned above reported all six subgroups also for the entire study period. For Asian Indians, Koreans, and Vietnamese, the adoption of the standard was evident in the trends for some states, however most of the affected states had very low mortality counts (<100 aggregate Asian deaths per year).

## Discussion

Asian Americans have been traditionally thought of as the “model minority” with higher socioeconomic status and education levels, and with lower mortality rates compared to NHWs.[5, 20] National vital statistics have reported that cancer is the leading cause of death among all Asian Americans.[6] This study revealed that heart disease is the leading cause of death in some Asian American subgroups, particularly Asian Indians, Filipinos, and Japanese. Previous studies in the U.S. and abroad have demonstrated increased mortality from heart disease among Asian Indians, Filipinos, and Japanese, as confirmed in our analysis.[21–26] We recently published a separate analysis examining Asian Americans and cardiovascular disease mortality, finding high rates of death from ischemic heart disease in Asian Indians compared to other racial/ethnic groups.[27] Cancer mortality rates were higher among Chinese, Korean, and Vietnamese males. As a follow-up to this analysis, we are examining site-specific cancer mortality for all Asian subgroups in order to examine what types of cancer are contributing most to the excess mortality between subgroups.

Stroke was the third leading cause of death among nearly every Asian subgroup, as well as NHW females, suggesting a broader approach may be needed in treating or preventing risk factors for stroke. Hypertension, an important stroke risk factor, is more prevalent in certain Asian American subgroups such as Filipinos.[27–31] Influenza and pneumonia also rank higher overall among Asian American females and is the fourth leading cause of death among Chinese females and Japanese males. A recent report found that there were gaps in receiving preventive services for older Asian American adults, particularly those who have never received a pneumococcal vaccination—one of the largest disparities facing Asian Americans which continues to worsen.[32] Diabetes mellitus (diabetes) was the fourth and fifth leading cause of death among all Asian females, while eighth for NHW females. For males, diabetes ranked only slightly higher in Asian Indians and Filipinos, compared to NHWs. Diabetes prevalence was also found to be higher among all Asian American subgroups, particularly Asian Indians and Filipinos, compared to NHWs.[33] Prevention and treatment efforts for diabetes will continue to be important in Asian American subgroups, as these populations grow, mature, and age in the U.S. While chronic lower respiratory disease is the fourth leading cause of death in NHW females, it ranks lower for Asian American females, likely due to overall lower smoking rates.[34] However, chronic lower respiratory disease ranked higher in Chinese (4th), Filipino (4th), and Vietnamese (5th) males relative to all other Asian subgroups, possibly due to higher smoking rates in these populations.[35] Suicide is ranked among the leading causes of death for males but not for females, shedding light on not only racial/ethnic differences but also sex differences in mortality burden. We have noted a particularly high rate of suicide among Korean males, and other studies have shown high rates of suicide for Koreans in South Korea.[36–38]

We found that malignant neoplasms are the first or second leading cause of death for all Asian ethnicities. Compared to NHWs, studies have shown that Asian American subgroups have higher incidence of cancer stemming from infectious conditions, such as tumors of the liver (hepatitis-related), stomach (h. pylori-related), and cervix (HPV-related), while they have lower incidence rates of lung, colorectal, breast, and prostate cancer.[11, 39, 40] However (although still lower than for NHWs) incidence rates for these latter cancer types do not reflect NHW trends in many Asian ethnicities: breast cancer incidence rates which are on the decline for NHWs are rising in all ethnicities except Japanese [40], possibly reflecting changes in population reproductive factors and rates of obesity; and there have been sharp increases in colorectal cancer among Koreans, Vietnamese, and Filipina and South Asian women [40], potentially attributable to risk factors such as obesity, lack of physical activity, and alcohol consumption. Unlike nationwide trends, smoking prevalence does not seem to be declining among Asian Americans, and their lung cancer incidence rates do not reflect decreasing trends detected for NHWs in recent decades, with increasing trends in South Asian men, and Filipina and Korean women [40]. Compliance with screening for early detection of breast, cervical and colorectal cancers is generally lower for Asians than NHWs; this is especially pronounced in foreign-born and low socioeconomic status populations [41–43], but has also been detected in fully insured populations in high income metropolitan areas.[44] Rising incidence rates in liver cancer (which are already higher in Asians than NHWs) reflect a need for improvement in hepatitis B vaccination coverage, especially in new immigrant populations [45].

Age-adjusted mortality rates and ratios are overall higher for NHWs compared to Asian subgroups (Table 4), as would be expected given the younger and largely healthy immigrant Asian populations. New immigrants are regularly screened for tuberculosis, syphilis, mental illness and other medical conditions.[46] Given that most Asian subgroups (except Japanese) are largely comprised of new immigrants (Table 1), lower relative mortality rates compared to NHWs should be expected among these groups due to self-selection into migration and the rigorous screening and selection process. For those with end-stage renal disease, previous data has suggested a possible survival advantage for Asian Americans over NHWs, most likely attributable to the effects of lower body mass index (BMI).[47] However, increasing mortality trends over time among certain Asian subgroups may also be due to new immigrant risk factors. It has been well documented that immigrant populations are faced with many sociocultural barriers to care (i.e. lack of access to health care, language barriers, and differing religious/cultural beliefs), which may lead to increased disease incidence, prevalence, and mortality.[48, 49] Observed increasing rates for stroke mortality among Asian Indians raises concern, and is consistent with a separate study that also found this paradoxical increase among Asian Indians compared to other racial/ethnic groups.[50] The study also found gaps in prevention strategies such as pharmacological treatment of risk factors, culturally competent policies, and lifestyle interventions to limit salt consumption, reduce tobacco use, and encourage physical exercise; all of which are known to reduce heart disease and stroke events.[51]

Immigration histories and acculturation may also play an important role for varying mortality causes among Asian American subgroups. Acculturation has been associated with the development of heart disease and unfavorable changes in heart disease risk factors among Chinese and Japanese Americans.[3, 52, 53] The Ni-Hon-San study is one of the most comprehensive studies of immigration, acculturation, and heart disease risk in Asians and suggests that environmental factors determine certain health outcomes for Japanese males. The study was initiated in 1965 to verify differences in heart disease risk factors in contrasting environments among Japanese males living in Japan (Hiroshima and Nagasaki) and the U.S. (Honolulu, Hawaii and San Francisco, California) and showed that coronary artery disease (CAD) and stroke mortality rates in Hawaii were intermediate between the high rates of stroke in Japan and the high rates of CAD in California.[54] Thus, it is important to understand the unique immigration and acculturation history of the varied Asian American subgroups to better predict disease morbidity and mortality over time. Additionally, the Ni-Han-San results are suggestive of environmental predictors of mortality risk among Asians, which is a separate set of research questions we hope to disentangle through linkages of county-specific socioeconomic and demographic data to our mortality database.

Potential limitations of our study include data based on death certificates, which may contain errors in the documented cause of death and race/ethnicity classification. Many studies have tested the reliability of race on death certificates by comparing it with self-reported race on separate data collection instruments such as the census or surveys.[55–58] Race/ethnicity misclassification on death records arises due to inconsistency in reporting race information. At times, race/ethnicity information may be provided to the funeral director by an informant (i.e. relative or friend), and if an informant is absent, by observation. Studies have shown that individuals who self-report as Asian on the census were sometimes reported as white on the death certificate, causing an underestimation of deaths and death rates for races other than white and black.[57, 58] Misclassification on death certificates between Asian subgroups is also likely and has been acknowledged as a limitation to reporting accurate characterizations of mortality burden.[59, 60] The degree of misclassification between each of the subgroups on death certificates is currently unknown and warrants further study. Recently, an NCHS study found that misreporting of Asian race/ethnicity leads to an underreporting of Asian death rates by approximately 7%.[56] In contrast, the U.S. Census race and ethnicity classification (denominator data) is based on self-report and is less subject to this type of misclassification. Additionally, some Asian Americans are likely to identify with multiple Asian subgroups and/or another race.[61] However, according to both the 2000 and 2010 U.S. censuses, this group represented only 1.7% of the entire Asian American population.

A separate limitation is the gradual adoption of the 2003 revision of the U.S. Standard Certificate of Death that encourages explicit reporting of Asian subgroup race/ethnicity. As of 2011, 36 states and the District of Columbia had implemented the 2003 revision of the U.S. Standard Certificate of Death. In our sensitivity analysis, we identified some states with differential reporting of Asian Indians, Koreans, and Vietnamese deaths’ by year, but, due to small populations of these ethnicities in the affected states, we suspect this would minimally affect our results. Regardless, mortality trends over time in these subgroups should be interpreted with caution as they may be slightly exaggerated due to gradual uptake of the new death certificate over the study period. Lastly, our presented analysis only considers underlying cause of death (as opposed to all listed causes of death).

While this analysis helps lay a foundation for understanding health disparities among Asian American subgroups, much more research needs to be done. As summarized in the 2010 “Call to Action” for heart disease in Asian Americans, recommendations for future studies include: changing existing data collection to include at least the 6 largest Asian American subgroups, developing standard measurement tools (widely applicable and comprehensive acculturation instruments and culturally-tailored food frequency questionnaires), and developing new research studies utilizing disaggregated data.[3] There also needs to be further examination of treatment patterns, risk prediction models, culturally specific lifestyle and medical interventions, and biological and social factors that impact mortality in Asian American subgroups. Our findings have warranted several new questions regarding Asian subgroup mortality. Study investigators are currently using the uniquely harmonized dataset (i.e. mortality, census, and ACS data) to examine the following topics of interest: examining site-specific cancer mortality to report which cancers impose the greatest threat of mortality among subgroups; evaluating whether nativity (foreign-born vs. U.S born) influences cause-specific mortality and survival within each subgroup; comparing cause-specific mortality of each subgroup to that of their respective Asian countries of origin; and testing which socioeconomic factors at the county level help explain mortality and survival differences among Asian American subgroups.

To our knowledge, no studies have previously reported the leading causes of death among diverse Asian American subgroups at the national level. Our findings show heterogeneity in the leading causes of mortality among Asian American subgroups that would otherwise be masked when these diverse subgroups are aggregated. These findings suggest that Asian American subgroups have unique disease risks, underscoring the need for further study and separation of subgroups on national health surveys. With additional research, culturally competent and cost-effective approaches can be tailored to optimize strategies in management and prevention of the leading causes of death among these rapidly growing ethnic populations.

## Supporting Information

S1 Table Total number of deaths, age-adjusted mortality rates, and rate ratios (RR) from all causes by racial/ethnic group and sex in the United States, 2003–2011 (50 States and District of Columbia). (DOCX)

S2 Table Rankings (Rk), death count, and percentage of death due to cause (%) by racial/ethnic group and sex, from 2003–2011 (50 States and District of Columbia). (DOCX)

S3 Table Age-adjusted (AR) mortality rates per 100,000 population by leading causes of death, racial/ethnic group, and sex: 50 U.S. States and District of Columbia, 2003–2011 yearly averages. (DOCX)
