# Supplementary material for: Leading Causes of Death among Asian American Subgroups (2003–2011)
Source: PLoS One. 2015 Apr 27;10(4):e0124341. doi: 10.1371/journal.pone.0124341 (PMC4411112; doi:10.1371/journal.pone.0124341)
Supplement: S1 Table — (DOCX) [file pone.0124341.s001.docx]

| **Female** | **NHW** | **Aggregate Asian** | **Asian Indian** | **Chinese** | **Filipino** | **Japanese** | **Korean** | **Vietnamese** |
| --- | --- | --- | --- | --- | --- | --- | --- | --- |
| Total # Deaths | 9,036,373 | 150,484 | 13,592 | 42,641 | 36,464 | 31,872 | 15,354 | 10,561 |
| Population Size | 73,030,653 | 4,908,967 | 976,037 | 1,375,599 | 1,084,108 | 300,131 | 579,867 | 593,225 |
| Adjusted mortality rate | 665.9 | 337.4 | 294.8 | 324.8 | 357.7 | 381.3 | 332.2 | 285.2 |
| Adjusted Rate Ratio | 1.00 | 0.51 | 0.44 | 0.49 | 0.54 | 0.57 | 0.49 | 0.43 |
| **Male** | **NHW** | **Aggregate Asian** | **Asian Indian** | **Chinese** | **Filipino** | **Japanese** | **Korean** | **Vietnamese** |
| Total # Deaths | 8,646,266 | 159,495 | 19,972 | 47,472 | 37,984 | 26,782 | 13,530 | 13,755 |
| Population Size3 | 70,544,365 | 4,429,486 | 1,064,888 | 1,232,121 | 863,474 | 219,570 | 476,646 | 572,786 |
| Adjusted mortality rate | 934.3 | 483.2 | 383.9 | 455.1 | 572.1 | 581.6 | 432.3 | 380.1 |
| Adjusted Rate Ratio | 1.00 | 0.52 | 0.42 | 0.49 | 0.61 | 0.62 | 0.46 | 0.41 |
| ^1^ Interpolated 2007 counts calculated from 2000 and 2010 U.S. Census Data; ^2^ Rates based on yearly averages, age adjusted to 2000 U.S standard population, and per 100,000 population | | | | | | | | |

S1 Table. Total number of deaths, age-adjusted mortality rates, and rate ratios (RR) from all causes by racial/ethnic group and sex in the United States, 2003-2011 (50 States and District of Columbia).
