# Supplementary material for: Leading Causes of Death among Asian American Subgroups (2003–2011)
Source: PLoS One. 2015 Apr 27;10(4):e0124341. doi: 10.1371/journal.pone.0124341 (PMC4411112; doi:10.1371/journal.pone.0124341)
Supplement: S2 Table — (DOCX) [file pone.0124341.s002.docx]

| **Female (A)** | **NHW** | | | **Aggregate Asian** | | | **Asian Indian** | | | **Chinese** | | | **Filipino** | | | **Japanese** | | | **Korean** | | | **Vietnamese** | | |
| --- | --- | --- | --- | --- | --- | --- | --- | --- | --- | --- | --- | --- | --- | --- | --- | --- | --- | --- | --- | --- | --- | --- | --- | --- |
| **Cause of Death** | **Rk** | **Death**  **Count** | **%** | **Rk** | **Death**  **Count** | **%** | **Rk** | **Death**  **Count** | **%** | **Rk** | **Death**  **Count** | **%** | **Rk** | **Death**  **Count** | **%** | **Rk** | **DeathCount** | **%** | **Rk** | **DeathCount** | **%** | **Rk** | **DeathCount** | **%** |
| **All Causes** |  | 9036373 | 100 |  | 150484 | 100 |  | 13592 | 100 |  | 42641 | 100 |  | 36464 | 100 |  | 31872 | 100 |  | 15354 | 100 |  | 10561 | 100 |
| **Diseases of heart** | 1 | 2307901 | 25.5 | 2 | 34921 | 23.2 | 1 | 3770 | 27.7 | 2 | 9941 | 23.3 | 2 | 8856 | 24.3 | 2 | 7032 | 22.1 | 2 | 3305 | 21.5 | 2 | 2017 | 19.1 |
| **Malignant neoplasms** | 2 | 1971845 | 21.8 | 1 | 41740 | 27.7 | 2 | 3052 | 22.5 | 1 | 12578 | 29.5 | 1 | 10360 | 28.4 | 1 | 8177 | 25.7 | 1 | 4624 | 30.1 | 1 | 2949 | 27.9 |
| **Cerebrovascular diseases** | 3 | 608974 | 6.7 | 3 | 13950 | 9.3 | 3 | 987 | 7.3 | 3 | 3987 | 9.4 | 3 | 3631 | 9.9 | 3 | 2880 | 9.0 | 3 | 1329 | 8.7 | 3 | 1136 | 10.8 |
| **Chronic lower respiratory diseases** | 4 | 565382 | 6.3 | 8 | 3734 | 2.5 | 7 | 334 | 2.5 | 8 | 1019 | 2.4 | 7 | 950 | 2.6 | 8 | 826 | 2.6 | 7 | 357 | 2.4 | 8 | 248 | 2.3 |
| **Alzheimer’s Disease** | 5 | 424930 | 4.7 | 7 | 3942 | 2.6 | 11 | 157 | 1.2 | 7 | 1113 | 2.6 | 9 | 699 | 1.9 | 4 | 1359 | 4.3 | 8 | 328 | 2.1 | 7 | 286 | 2.7 |
| **Accidents-Unintentional injuries** | 6 | 311495 | 3.4 | 6 | 4935 | 3.3 | 5 | 509 | 3.7 | 6 | 1360 | 3.2 | 5 | 1075 | 2.9 | 7 | 928 | 2.9 | 4 | 645 | 4.2 | 4 | 418 | 3.9 |
| **Influenza and pneumonia** | 7 | 230580 | 2.6 | 5 | 5037 | 3.3 | 6 | 353 | 2.6 | 4 | 1756 | 4.1 | 6 | 1021 | 2.8 | 5 | 1019 | 3.2 | 6 | 575 | 3.7 | 6 | 313 | 2.9 |
| **Diabetes Mellitus** | 8 | 223091 | 2.5 | 4 | 5660 | 3.8 | 4 | 648 | 4.8 | 5 | 1419 | 3.3 | 4 | 1675 | 4.6 | 6 | 934 | 2.9 | 5 | 581 | 3.8 | 5 | 403 | 3.8 |
| **Nephritis, nephrotic syndrome, and nephrosis** | 9 | 154038 | 1.7 | 9 | 2759 | 1.8 | 8 | 243 | 1.8 | 9 | 755 | 1.8 | 8 | 874 | 2.4 | 9 | 467 | 1.5 | 9 | 224 | 1.5 | 9 | 196 | 1.9 |
| **Septicemia** | 10 | 128110 | 1.4 | 11 | 1608 | 1.0 | 9 | 198 | 1.5 | 11 | 373 | .87 | 11 | 471 | 1.3 | 11 | 329 | 1.0 | 12 | 121 | .79 | 12 | 116 | 1.1 |

S2 Table. Rankings (Rk), death count, and percentage of death due to cause (%) by racial/ethnic group for females (A) and males (B), from 2003-2011 (50 States and District of Columbia).

| **Male (B)** | **NHW** | | | **Aggregate Asian** | | | **Asian Indian** | | | **Chinese** | | | **Filipino** | | | **Japanese** | | | **Korean** | | | **Vietnamese** | | | |
| --- | --- | --- | --- | --- | --- | --- | --- | --- | --- | --- | --- | --- | --- | --- | --- | --- | --- | --- | --- | --- | --- | --- | --- | --- | --- |
| **Cause of Death** | **Rk** | **Death**  **Count** | **%** | **Rk** | **Death**  **Count** | **%** | **Rk** | **Death**  **Count** | **%** | **Rk** | **Death**  **Count** | **%** | **Rk** | **Death**  **Count** | **%** | **Rk** | **Death**  **Count** | **%** | **Rk** | **Death**  **Count** | **%** | **Rk** | **Death**  **Count** | **%** |  |
| **All Causes** |  | 8646266 | 100 |  | 159495 | 100 |  | 19972 | 100 |  | 47472 | 100 |  | 37984 | 100 |  | 26782 | 100 |  | 13530 | 100 |  | 13755 | 100 |  |
| **Diseases of heart** | 1 | 2311423 | 26.8 | 2 | 40050 | 25.1 | 1 | 6234 | 31.2 | 2 | 10943 | 23.1 | 1 | 10733 | 28.3 | 1 | 7057 | 26.3 | 2 | 2627 | 19.4 | 2 | 2456 | 17.9 |  |
| **Malignant neoplasms** | 2 | 2144938 | 24.8 | 1 | 43880 | 27.5 | 2 | 3602 | 18.0 | 1 | 15124 | 31.9 | 2 | 9526 | 25.1 | 2 | 6588 | 24.6 | 1 | 4592 | 33.9 | 1 | 4448 | 32.3 |  |
| **Chronic lower respiratory diseases** | 4 | 500307 | 5.8 | 5 | 6089 | 3.8 | 8 | 442 | 2.2 | 4 | 2092 | 4.4 | 4 | 1697 | 4.5 | 7 | 870 | 3.2 | 8 | 425 | 3.1 | 5 | 563 | 4.1 |  |
| **Accidents-Unintentional injuries** | 3 | 513818 | 5.9 | 4 | 7411 | 4.6 | 3 | 1307 | 6.5 | 6 | 1937 | 4.1 | 6 | 1520 | 4.0 | 5 | 1026 | 3.8 | 4 | 809 | 5.9 | 4 | 812 | 5.9 |  |
| **Cerebrovascular diseases** | 5 | 383248 | 4.4 | 3 | 11337 | 7.1 | 4 | 1032 | 5.2 | 3 | 3406 | 7.2 | 3 | 3026 | 7.9 | 3 | 1952 | 7.3 | 3 | 838 | 6.2 | 3 | 1083 | 7.9 |  |
| **Diabetes Mellitus** | 6 | 232232 | 2.7 | 6 | 5565 | 3.5 | 5 | 939 | 4.7 | 7 | 1310 | 2.8 | 5 | 1588 | 4.2 | 6 | 906 | 3.4 | 7 | 451 | 3.3 | 7 | 371 | 2.7 |  |
| **Intentional self-harm (suicide)** | 7 | 208005 | 2.4 | 8 | 3443 | 2.2 | 6 | 575 | 2.9 | 8 | 813 | 1.7 | 9 | 609 | 1.6 | 11 | 390 | 1.4 | 5 | 658 | 4.9 | 6 | 398 | 2.9 |  |
| **Influenza and pneumonia** | 8 | 187979 | 2.2 | 7 | 5465 | 3.4 | 7 | 479 | 2.4 | 5 | 2044 | 4.3 | 7 | 1097 | 2.9 | 4 | 1039 | 3.9 | 6 | 464 | 3.4 | 8 | 342 | 2.5 |  |
| **Alzheimer’s Disease** | 9 | 177508 | 2.1 | 10 | 1998 | 1.3 | 13 | 130 | 0.65 | 10 | 602 | 1.3 | 11 | 372 | 0.98 | 8 | 616 | 2.3 | 10 | 139 | 1.0 | 12 | 139 | 1.0 |  |
| **Nephritis, nephrotic syndrome, and nephrosis** | 10 | 150596 | 1.7 | 9 | 2808 | 1.8 | 9 | 348 | 1.7 | 9 | 790 | 1.7 | 8 | 843 | 2.2 | 9 | 476 | 1.8 | 9 | 159 | 1.2 | 11 | 192 | 1.4 |  |
| Source: National Center for Health Statistics: Diseases of the heart (International Classification of Diseases- 10^th^ revision [ICD-10] codes I00-I09, I11, I13, I20-I51); Malignant Neoplasms (C00-C97); Chronic lower respiratory diseases (J40-J47); Accidents-unintentional injury (V01-X59, Y85-Y86); Cerebrovascular disease (I60-I69); Diabetes Mellitus (E10-E14); Intentional self-harm (suicide) (U03, X60-X84, Y87.0); Influenza and pneumonia (J09-J18); Alzheimer’s Disease (G30); Nephritis, nephrotic syndrome, and nephrosis (N00-N07, N17-N19, N25-N27); Septicemia (A40-A41). | | | | | | | | | | | | | | | | | | | | | | | | |  |
