# Supplementary material for: Leading Causes of Death among Asian American Subgroups (2003–2011)
Source: PLoS One. 2015 Apr 27;10(4):e0124341. doi: 10.1371/journal.pone.0124341 (PMC4411112; doi:10.1371/journal.pone.0124341)
Supplement: S3 Table — (DOCX) [file pone.0124341.s003.docx]

| **Female** | **NHW** | **Aggregate Asian** | **Asian Indian** | **Chinese** | **Filipino** | **Japanese** | **Korean** | **Vietnamese** |
| --- | --- | --- | --- | --- | --- | --- | --- | --- |
| **Cause of Death** | **AR** | **AR** | **AR** | **AR** | **AR** | **AR** | **AR** | **AR** |
| Diseases of the heart | 159.1 | 82.3 | 96.5 | 77.9 | 93.7 | 77.8 | 81.5 | 62.6 |
| Malignant neoplasms | 155.9 | 85.9 | 52.2 | 90.9 | 86.9 | 106.7 | 84.9 | 65.6 |
| Cerebrovascular diseases | 41.3 | 31.9 | 25.2 | 30.9 | 36.2 | 32.8 | 30.1 | 32.4 |
| Chronic lower respiratory diseases | 42.2 | 8.9 | 9.4 | 8.0 | 10.4 | 9.5 | 8.8 | 7.9 |
| Alzheimer’s Disease | 27.3 | 9.8 | 5.1 | 8.8 | 8.4 | 13.9 | 8.8 | 10.5 |
| Accidents-Unintentional injuries | 28.4 | 10.2 | 7.7 | 9.9 | 9.7 | 13.2 | 11.4 | 8.8 |
| Influenza and pneumonia | 15.8 | 12.1 | 9.1 | 13.8 | 11.4 | 10.9 | 14.7 | 10.5 |
| Diabetes Mellitus | 16.8 | 12.9 | 15 | 11.1 | 16.7 | 11 | 13.4 | 11.9 |
| Nephritis, nephrotic syndrome, and nephrosis | 10.9 | 6.4 | 6.0 | 5.9 | 8.9 | 5.4 | 5.3 | 5.7 |
| Septicemia | 9.5 | 3.7 | 4.6 | 2.9 | 4.8 | 3.9 | 2.7 | 3.3 |
| **Male** | **NHW** | **Aggregate Asian** | **Asian Indian** | **Chinese** | **Filipino** | **Japanese** | **Korean** | **Vietnamese** |
| **Cause of Death** | **AR** | **AR** | **AR** | **AR** | **AR** | **AR** | **AR** | **AR** |
| Diseases of heart | 250.1 | 126.4 | 131 | 108.5 | 167.4 | 149.2 | 94.7 | 75.9 |
| Malignant neoplasms | 221.8 | 124.2 | 65.2 | 135.9 | 131.6 | 145.8 | 132.9 | 108.8 |
| Chronic lower respiratory diseases | 53.9 | 21.4 | 12.6 | 21.9 | 30.2 | 18.3 | 18.1 | 21.5 |
| Accidents-Unintentional injuries | 57.9 | 18.5 | 15.0 | 16.9 | 19.1 | 25.8 | 18.9 | 16.4 |
| Cerebrovascular diseases | 42.5 | 36.2 | 24.3 | 33.6 | 46.9 | 40.8 | 28.5 | 33.52 |
| Diabetes Mellitus | 24.3 | 16.8 | 18.9 | 12.7 | 22.8 | 19.6 | 15.7 | 11.5 |
| Intentional self-harm (suicide) | 22.9 | 7.1 | 4.9 | 6.2 | 6.3 | 11.0 | 13.5 | 6.3 |
| Influenza and pneumonia | 21.3 | 19.6 | 13.2 | 21.8 | 19.2 | 21.2 | 20.6 | 13.8 |
| Alzheimer’s Disease | 20.8 | 7.8 | 5.1 | 6.7 | 7.6 | 12.1 | 6.8 | 6.5 |
| Nephritis, nephrotic syndrome, and nephrosis | 16.7 | 9.3 | 8.7 | 8.1 | 13.7 | 10.0 | 5.6 | 6.4 |
